# The origins of California’s gun violence restraining order law: a case study using Kingdon’s multiple streams framework

**DOI:** 10.1186/s12889-023-16043-6

**Published:** 2023-06-30

**Authors:** Elizabeth A. Tomsich, Veronica A. Pear, Julia P. Schleimer, Garen J. Wintemute

**Affiliations:** 1 Violence Prevention Research Program, Department of Emergency Medicine, University of California Davis, 4301 X St., Sacramento, CA 95817 USA; 2 California Firearm Violence Research Center, University of California Davis, 4301 X St., Sacramento, CA 95817 USA

**Keywords:** Violence, Firearm policy, Multiple streams framework

## Abstract

**Background:**

Firearm violence is a major public health problem in the United States, yet most states lack a mechanism to temporarily remove firearms from individuals who are at high and imminent risk of harming themselves or others and are not otherwise prohibited. Extreme risk protection order (ERPO) laws are intended to close this gap. The current study examines the passage of California’s gun violence restraining order (GVRO) bill using Kingdon’s multiple streams framework.

**Methods:**

This study was based on an analysis of interview data from six key informants involved in the passage of the GVRO legislation.

**Results:**

Findings indicate policy entrepreneurs framed the problem and designed the policy to target individuals at behavioral risk of imminent firearm violence. Policy entrepreneurs comprised an integrated policy network that engaged in a lengthy period of collaboration and bargained with interest groups to yield a bill that satisfied diverse concerns.

**Conclusions:**

This case study may inform efforts in other states to pass ERPO policies and other firearm safety laws.

**Supplementary Information:**

The online version contains supplementary material available at 10.1186/s12889-023-16043-6.

## Background

Interpersonal and self-directed firearm violence account for a substantial proportion of fatal and non-fatal injuries in the United States, yet firearm violence prevention laws remain contentious. In 2020, firearm-related injuries were among the top five causes of death for people between 1–44 years of age and the leading cause of death for youth 1–24 years of age; the prevalence of nonfatal firearm injuries is twice that of fatalities [1–3]. While mass shootings account for less than 1% of all U.S. firearm homicides [4], international comparisons indicate the country has the largest share of public mass shooters [5]. The number of firearms—1.2 per civilian–similarly outpaces that of any other nation by a large margin [6]. Despite support for proposals restricting firearm access for people determined to be a danger to themselves or others [7], most states lack a mechanism to temporarily recover firearms from such individuals.

Extreme risk protection order (ERPO) laws are intended to close this gap. Following early ERPO laws using risk warrant mechanisms in Connecticut (1999) and Indiana (2005), California was the first state to pass an ERPO law based on restraining orders in 2014. While risk warrant laws permit law enforcement to temporarily recover firearms through a warrant-based procedure, ERPO laws allow law enforcement and other eligible petitioners to file for a civil court order that temporarily restricts a respondent’s access to firearms. Unlike risk warrant laws, ERPO policies may be used to prevent the acquisition of a firearm by a non-owner. California’s restraining order model has been adopted by all subsequent states enacting an ERPO law. In California, ERPOs are called gun violence restraining orders (GVROs). GVROs authorize civil courts to temporarily prohibit the purchase and possession of firearms and ammunition by persons who exhibit dangerous or threatening behaviors, have or could have access to a firearm, and are not subject to an existing firearm prohibition. Lawmakers considering ERPO laws may view states such as California as “policy laboratories” and seek to understand the history behind the passage of the GVRO law to replicate its success.

Formal consideration of California’s GVRO law began with the mass shooting in Isla Vista, California in 2014, and was quickly followed by approval by the legislature. The attack, perpetrated by a self-described involuntary celibate or “incel,” killed six people and injured 14 others near the University of California, Santa Barbara campus. However, connections among the coalition of lawmakers, legislative staff, and activists responsible for passing the law, as well as efforts to raise awareness and garner support for an ERPO law in California, began over a decade prior. Without documenting this history, we lack a complete account of the strategies, challenges, and facilitators related to the GVRO law’s approval.

Recent work stemming from an early-stage evaluation of the law presents information on its implementation and outcomes [8–14]. The current study contributes to this literature by providing the history behind the GVRO policy’s enactment–a rare account of the successful passage of a state-level firearm safety law. Specifically, the study describes the key actors, networks, events, and efforts behind the bill’s development between 2001 and its approval in 2014. We use Kingdon’s multiple streams policy framework to describe how largely “hidden” policy entrepreneurs, some with histories of tragedy due to firearm violence, defined the problem, proposed a solution, and capitalized on a window of opportunity that led to the successful adoption of the GVRO law.

## Methods

This study used interview data from six key informants involved in the passage of the GVRO legislation. Key informants were identified through professional relationships with the authors, prior informant interviews, and by recommendation from other participants. Key informants comprised four policy advocates, a clinical professor of psychiatry, and a professor of emergency medicine. Two of the policy advocates worked for separate firearm violence prevention organizations, while the other two worked for a variety of legislative offices. All key informants recruited to the study accepted the invitation to participate. An investigator (ET) conducted interviews inquiring about participants’ experience with the policy, other persons involved, perceptions about the success of various strategies, and factors that facilitated or impeded the bill’s passage. Interview length ranged from 30 min to an hour. Four interviews were audio recorded and transcribed; two interviews were documented by the interviewer’s notes. One investigator (ET) reviewed the interviews to develop a timeline, identify patterns in the data, and generate higher-order themes, which were based on multiple streams theory and iteratively updated throughout the process. Each participant had the opportunity to review the manuscript, modify their quotes, and provide feedback on the authors’ interpretation of the findings. In addition to interviews, we reviewed media and legislative hearings documents.

**Fig. 1:**
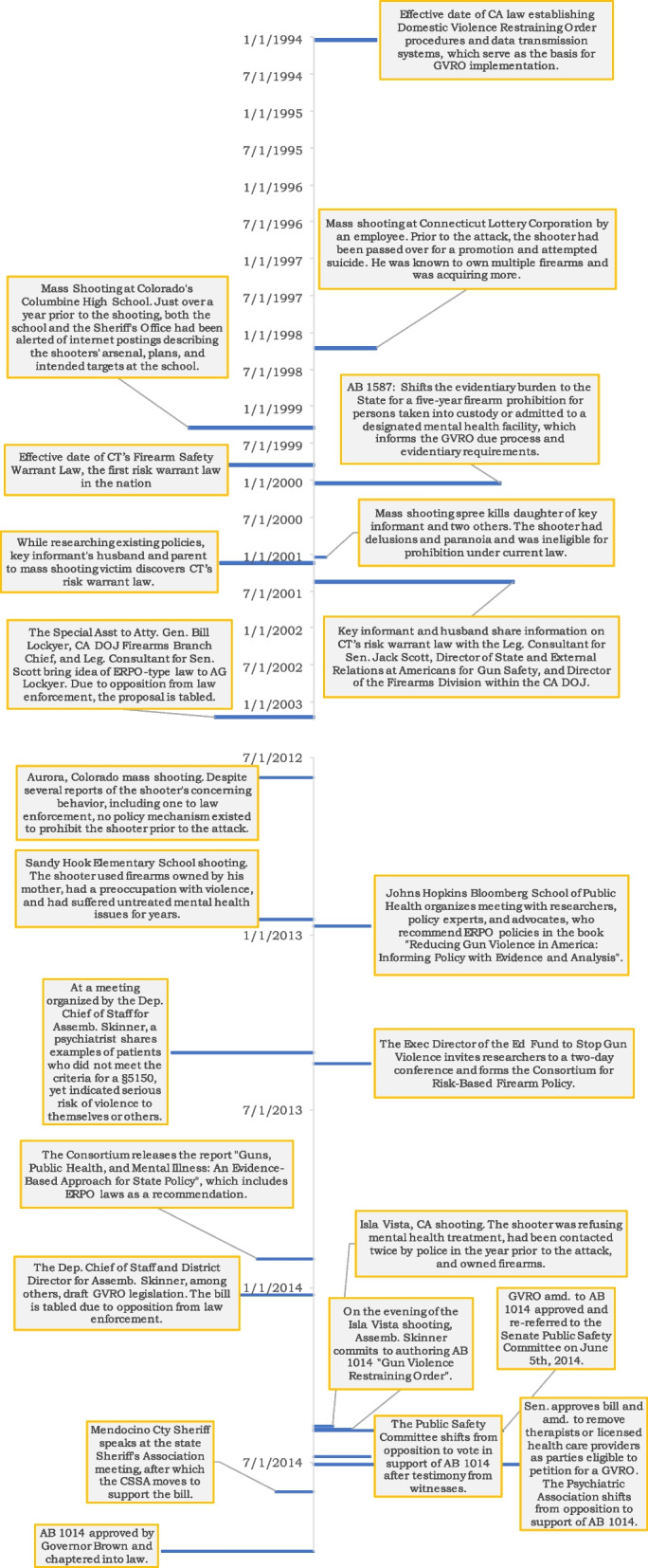
GVRO Timeline

We applied Kingdon’s multiple streams framework, which has been used to examine firearm policies in other work [15–17], to organize themes, strategies, events, and policy stages and facilitate comparison within the policy science literature. Kingdon’s [18] framework considers policymakers as limited by bounded rationality, making decisions under the constraints of incomplete information and ambiguity in the definition or framing of a problem. Rather than a linear process wherein a policymaker identifies a problem, the bureaucracy and policy advocates generate solutions, and the policymaker selects the ideal proposal, Kingdon [18] supposed that the problem, policy, and politics operate as distinct streams, proceeding in any order. Once a problem has been coupled with a solution and the political context is favorable toward action, a policy window opens, allowing the proposed agenda to be accepted. In the following sections, we describe the problem stream, policy stream, policy window, and politics stream, detailing how the problem and policy streams coupled to result in the adoption of the GVRO law. Each stream and corresponding barriers and facilitators are summarized in Supplementary Table 1. Figure 1 presents a timeline of events.

## Results

### Problem stream

Kingdon’s [18] problem stream refers to the social construction of societal conditions as a significant problem requiring political intervention. Factors influencing the problem stream include the magnitude of the problem, limitations of existing policy, feedback to legislators, and focusing events—high-profile and/or frequent incidents that bring the problem to the attention of lawmakers and the public.

#### Indicators of the problem

The scale of firearm violence in the United States is profound; firearm suicide and homicide accounted for 47,286 deaths in 2021, and in 2013, the year prior to the enactment of the GVRO bill, there were 32,383 deaths [19]. Suicides comprised the majority of firearm-related deaths (56% in 2021, 65% in 2013) [19]. Firearm violence of all kinds has broad negative consequences, not only for those who die, but also for surviving victims [20], witnesses [21, 22], and communities [23].

#### Focusing events

Mass shootings represent a small proportion of all US firearm homicides [4] but have a disproportionate impact on Americans’ perceptions of and concerns about firearm violence. Approximately 1 in 3 Americans report avoiding places and events out of fear of a mass shooting [24]. Mass shootings serve as focusing events, drawing attention to the problem of firearm violence. They “reinforce some preexisting perception of a problem” and “focus attention on a problem that was already ‘in the back of people’s minds’” (p. 98) [25].

One key informant noted that prior to the mass shooting in Sandy Hook, Connecticut, public advocacy surrounding gun violence prevention looked very different: “The horrific nature of seeing 20 children and six educators killed in that way I think shook the nation awake.” This focusing event spurred widespread outrage over legislative inaction, providing feedback to legislators. Another key informant reported that “Sandy Hook happened in December 2012, Isla Vista was Memorial Day 2014, and […] people’s nerves […] were still frayed, they were still upset that nothing happened in Congress.”

#### Effectiveness of existing policy

Evidence on existing firearm violence prevention policies has been limited and/or mixed, but some policies have been found to be preventive of violence such as minimum age requirements, child-access prevention laws, prohibitions associated with domestic violence and misdemeanor violent crime, and the surrender of firearms by prohibited owners or possessors [26–29]. Others have been found to confer risk, such as concealed carry and stand-your-ground laws [26]. Although California established a relatively broad number of firearm violence prevention policies prior to the GVRO law, gaps in existing mechanisms to restrict access to firearms for individuals exhibiting dangerous or threatening behaviors remained. For example, many firearm prohibitions, such as those resulting from convictions or protective orders, may not be adequately enforced [30] or widely used, e.g., because they require a protected party to serve as a petitioner. Likewise, involuntary psychiatric hospitalizations, which result in a prohibition, require dangerousness to be a result of a mental illness, yet mental illness is not the source of dangerousness for many people who pose a threat to themselves or others [31, 32].

While evidence on ERPO laws was limited prior to the GVRO law being adopted, one evaluation of Connecticut’s risk warrant law was available at the time that California was considering its proposal. This law permitted a state’s attorney and any two police officers to petition a judge for approval to recover firearms and/or ammunition when there was probable cause to believe that a person was at imminent risk of injuring themselves or others and possessed a firearm (as of 2021, family, household members, and medical professionals can also serve as petitioners). It found that a minority of respondents to these orders presented with a history of involuntary hospitalization, receipt of services from the Department of Mental Health and Addiction Services, or active psychiatric treatment involvement [33]. Almost 80% of those who had a firearm recovered by law enforcement had no history of a diagnosed mental illness, and fewer than 1% were in treatment at the time of recovery.

#### Definition of the problem

All key informants pointed to the problem of the absence of an adequate mechanism to remove firearms quickly and temporarily from individuals at high risk of violence. Informants, many of whom had experienced personal tragedies—including loss from mass shootings or suicides—understood that such a mechanism could help prevent multiple forms of firearm violence.

One key informant reported that, while the prevention of mass shootings makes ERPO laws appealing to the public and lawmakers, “I think that, for me, the stories of suicides being prevented are just as every bit powerful, meaningful, and are having such an incredible impact in communities around the country, that both of those prevention models should be celebrated.” Another key informant, speaking on ERPO laws and the loss of his friend from firearm suicide, stated:

*I wish I had more tools. I wish I knew more. You know, now I would have done something totally different in so many ways. It's really important, because when I think of people who have a loved one or a relative they care about where they're worried about suicidality, I want them to have this tool.*

A key informant who works in emergency medicine recalled speaking with a psychiatrist colleague about the need for interventions for interpersonal violence and individuals in acute crisis:

*When we got together to talk about the idea and draft language, the interest was broadly in the ability to prevent firearm violence. [My colleague] and I were making the case […] about all the people who clearly are time bombs about to go off, but don’t meet criteria for psychiatric holds, haven’t committed a crime, [and] there’s nothing we can do, a problem that had plagued my specialty and hers since forever.*

#### Problem framing

The multiple streams framework asserts that the existence of varied problem frames—each of which informs the understanding of an issue—creates ambiguity in policymaking [34]. In the case of the ERPO law, frequent discussion of mental illness in media coverage of mass shootings complicated the framing of the problem.

Policy entrepreneurs—persons “[…] responsible not only for prompting important people to pay attention, but also for coupling solutions to problems and for coupling both problems and solutions to politics” (p.21) [18]—sought to define the problem as behavioral rather than rooted in mental illness, emphasizing that mental illness primarily increases risk for violence victimization and not perpetration [35, 36]. One key informant perceived the media and groups opposed to firearm violence prevention policies as scapegoating individuals with mental illness, wrongly suggesting “that this country has a problem with mental illness and not a gun violence problem.” When multiple causes are attributed to a problem, such as firearm violence, advocates committed to maintaining the policy status quo can divert attention from firearm policy to other policy arenas, such as mental health care [15].

#### Feedback to legislators

Key informants reported that media coverage influenced the problem stream by amplifying the voices of firearm violence survivors:

*[…] survivors are always the most important voice, right? Survivors, individuals who have had their loved ones killed by suicide or by firearms. I think that always makes the biggest difference. […] I don't think that there's stronger voices in this than people who wish that they could have prevented these permanent tragedies that they've experienced from happening in the first place.*

However, media coverage often attributed mass shootings primarily to mental illness, complicating the messaging to legislators about the problem definition and corresponding policy solutions.

### Policy stream

The policy stream includes proposed ideas or policy solutions and the process of negotiation and debate over alternative ideas. Kingdon [18] described the policy stream as a “primeval soup” or evolutionary process where some ideas float to the top; typically, these are technically and economically feasible and consistent with values held by the public and lawmakers.

#### Policy network

Discussions of an ERPO law in California began almost a decade and a half prior its passage. In 2001, a mass shooting in Nevada County, California killed three people, one of whom was the daughter of a key informant. Although the shooter's family and a social worker attempted to have him hospitalized for delusions and paranoia prior to the shooting, the shooter—who was known to own multiple firearms—refused treatment and was not prohibited from firearm access. Already active in the firearm violence prevention movement via charitable donations, the key informant’s husband began researching firearm policies within a month of their daughter’s killing and found Connecticut’s Firearm Safety Warrant Law.

In the summer of 2001, the key informant and her husband shared information on Connecticut’s law with the Legislative Consultant for Senator Jack Scott; the Director of State and External Relations at Americans for Gun Safety; and the Director of the Firearms Division within the California Department of Justice, whom they first met while lobbying. At the urging of the key informant, the Legislative Consultant for Senator Jack Scott asked the California Senate Office of Research to write a report on similar laws in other states to identify options for California; the report reviewed Connecticut’s and Indiana’s risk warrant laws.

These early efforts exemplify the importance of relationships among policy entrepreneurs, which developed years prior to the GVRO bill. Several key informants met in the early 2000s while working on firearm violence prevention legislation, and two worked with Senator Jack Scott. One informant noted, “There was a group of people that worked on firearms policy in California for a long time,” and that relationships between stakeholders were strengthened by a history of collaboration in California, where an established group of advocates had worked together to put into place some of the most expansive firearm violence prevention policies in the nation.

These relationships constituted an integrated policy network; such networks are smaller than less integrated networks and involve collaboration, higher administrative capacity, and more restricted access for non-specialists without expertise [37]. Within this policy subsystem, problems and new (“alternative”) policies develop and undergo consensus-based debate before politicians become involved. Integrated networks tend to engage in a lengthy “softening up” period, where proposals are iteratively adapted, giving rise to an “emerging consensus” around a practicable policy alternative, followed by rapid uptake [37].

#### Policy framing

Misperceptions in the problem stream about mental illness and confusion over the need for an ERPO law within the context of existing policy, including mental health emergency hospitalizations (§5150 Welfare and Institutions Code), persisted in the policy stream. As the applicability of a policy to a particular problem varies by the definition of said problem, policy entrepreneurs needed to articulate how the issue of firearm access by persons at high risk of violence extended beyond situations involving—and policy mechanisms for—mental illness.

In the spring of 2013, after the mass shooting at Sandy Hook Elementary School in Newtown, Connecticut, the Deputy Chief of Staff for Assembly Member Nancy Skinner organized a meeting of stakeholders, including representatives from the California Psychiatric Association, Disability Rights California, the California Medical Association, Brady California, the Law Center to Prevent Gun Violence (now merged with Giffords Law Center to Prevent Gun Violence), the Coalition to Stop Gun Violence, and law enforcement. At the meeting, a psychiatrist reported that she often saw patients who were ineligible for a §5150 hold but displayed threatening or dangerous behavior, such as individuals who made explicit or implicit threats when intoxicated but were sober when the evaluation occurred. This psychiatrist, a key informant, described a number of misconceptions about §5150 holds:

*And what people failed to understand is, first of all, that the §5150 has to be due to a mental illness. So if a woman's delusional and believes that her children are angels, and they need to return to heaven, and she needs to kill them for that to happen, she is dangerous to them because of a mental illness. But if a woman wants to not be encumbered by her child anymore and wants to kill her child, that's not a mental illness; that's just really awful. We're not going to fix that, and that's not going to meet criteria for a §5150. […] So if somebody says, "I hate my old boss. He's such a jerk. I'm going to go shoot up my workplace," a §5150 's not going to do anything about that.*

One key informant reported this was the “missing piece of information” needed to articulate the policy gap filled by GVROs and to soften up those resistant to a policy solution focused on behavioral risks, rather than mental illness. Subsequently, at the meeting, the key informant and her husband re-introduced the idea of an ERPO and found, for the first time, a receptive audience. They had previously presented the idea of the Connecticut law at a California Firearm Policy Coalition meeting in 2012, where stakeholders representing legislative offices and firearm violence prevention advocacy groups articulated their feeling that there was no need for such a law within California’s existing domestic violence restraining order (DVRO), protective order, and 5150 prohibition framework. This marked the beginning of advocates coalescing around the goal of passing an ERPO law in California. After the 2013 meeting, policy entrepreneurs created a chart comparing GVROs to §5150, DVRO, and other protective order mechanisms. The chart was shared with legislative offices when lobbying for the bill to demonstrate how GVROs uniquely contributed to the existing policy landscape.

To respond to claims that firearm violence is a problem of mental illness rather than firearm access and behavioral risk, the Executive Director of the Coalition to Stop Gun Violence and Educational Fund to Stop Gun Violence (now the Johns Hopkins Center for Gun Violence Solutions) invited mental health and public health scholars, activists, practitioners, and law enforcement to a two-day conference in March 2013. Following this conference, the Executive Director, working with colleagues who attended the meeting, formed the Consortium for Risk-Based Firearm Policy. One key informant summarized the discussion at the meeting as follows:

*So, when [what would become] the Consortium for Risk-Based Firearm Policy met to tackle the issue of whether or not mental illness was a risk factor for violence, it became very clear, very quickly, that the answer was, "No, mental illness is not a significant risk factor. In fact, only four percent of violent acts in the country take place solely because of a mental illness." That night, when everybody had worked through, "Well, what do we do about the guns then?" – and there were a lot of different opinions – this idea of the gun violence restraining order is what came forward.*

At this meeting, key informants reported that a firearm violence researcher shared information on Connecticut’s risk warrant law. After the Virginia Polytechnic Institute and State University shooting in 2007, law enforcement realized risk warrants could be used to remove firearms from individuals making threats of interpersonal or mass violence, irrespective of mental illness, and uptake of this tool increased. Discussions of the risk warrant law led policy entrepreneurs away from solely considering mental health or criminal justice mechanisms to address the problem of firearm violence:

*[…] the idea came out of that meeting was what if instead of this red herring of mental illness […], we looked at where the actual risk lay, which is in behavioral risk factors? And what if we found a way to, if you were at this heightened risk of violent behavior – according to the evidence and the research that we have about what these indicators of risk are – what if we just found a system to just separate that individual from the firearm without creating new avenues of criminality, but simply, separating them from firearms?*

In late 2013, the Consortium released the report, "Guns, Public Health, and Mental Illness: An Evidence-Based Approach for State Policy," which recommended the adoption of ERPO laws. This echoed researchers, policy experts, and advocates who advocated for ERPO laws to prevent firearm violence in "Reducing Gun Violence in America: Informing Policy with Evidence and Analysis" following a meeting earlier in the year at the Johns Hopkins Bloomberg School of Public Health.

#### Softening the proposal

As Kingdon notes, policy entrepreneurs “[…] try out their ideas on others by going to lunch, circulating papers, publishing articles, holding hearings, presenting testimony, and drafting and pushing legislative proposals” (pp. 122–3) [18], as policy ideas undergo a “[…] process of consideration, floating up, discussion, revision, and trying out again” (p. 149) [18]. Once they published their first report, Consortium members provided a series of educational forums to lawmakers, activists, prosecutors, city attorneys, and law enforcement, including at Capitol Hill and in Los Angeles and San Francisco. This approach enabled advocates to “soften up” the proposal among stakeholders and legislators:

*My memories of this were, we aligned our allies in the way that made us really solid, so we spent a lot of time talking and listening and drafting and re-drafting. We didn’t go to the sheriffs and say, “Here’s the bill. Take it or leave it. Please endorse this.” It was a very iterative process, and then, once the [Isla Vista] shooting happened, we did these forums. One big forum in L.A. and one in San Francisco […]. We had legislators. We had over 100 really key stakeholders in each location, and we presented, they asked questions for an hour, and, once we were done, people felt there was an action plan, and they felt they were included in it. So bringing the academics to work with the grassroots and grasstops advocates is a lesson that I take forever from that campaign.*

Overall, there were few conflicts over alternative proposals. One informant noted, “I think the biggest barrier was ‘how do we align these interest groups on a new tool?’” Interest groups included medical and mental health groups, disability rights, law enforcement, and policy advocates.

#### Logistic and economic feasibility

Another element of the bill that facilitated support was its logistic and economic feasibility, as policy entrepreneurs used existing policies and policy infrastructures to inform its development. The Deputy Chief of Staff and District Director for Assembly Member Nancy Skinner, among others, modeled the GVRO bill on existing DVRO law, using similar procedures and due process requirements. The due process and evidentiary requirements were also informed by the January 2000 law, AB 1587, which shifted the evidentiary burden of the §5150 firearm prohibition from the individual to the State, which had to show by a preponderance of the evidence that the person would be unlikely to use firearms in a safe and lawful manner.

With a viable policy proposal established, the policy stream became “ripe,” or ready to be coupled with a problem [38].

### Policy window

When two or three streams merge, “policy windows” open, which increase the likelihood of adopting policies alternative to the status quo. In the case of the GVRO policy, agenda setting, alternative specification (the selection of one or a small set of viable policy alternatives available to address the problem), and decision making intersected on May 23, 2014, when a focusing event occurred: the Isla Vista mass shooting. The shooter refused mental health treatment and had been contacted twice by police in the year prior to the attack, once for an attempted assault and a second time for a welfare check regarding disturbing YouTube postings. The shooter had purchased one firearm seven months prior to the first contact, and a second two months before the second police contact. Two key informants identified the preventable nature of the attack as critical to its policy significance:

*[…] when you saw the details of that shooting, a young man who – his parents knew he was capable of doing what he did – called for the wellness check. Police didn't have the tools to remove the guns, even though his parents knew he was at risk of doing something exactly like he did. And it was just such a clear-cut sign that it was a shooting that could have been prevented.*

*It was kind of like after the Cleveland schoolyard shooting in Stockton in 1989. There was a very acute sense that something needs to be done. There was, I think, a widespread, at least among policymakers, recognition that we could have avoided this. Which was true and which was made really clear by the facts of the case, which were just impossible to walk away from. […] there was the memory of other mass shootings where declarations or some sort of advance notice was involved. Certainly Sandy Hook, where people knew there was a problem and didn’t do anything about it, etcetera. […]. Everybody wanted to have a way to prevent the next one.*

Media coverage provided feedback to legislators and increased the priority of firearm violence prevention on the policy agenda. One informant reported that media largely focused on the role of mental illness in mass shootings rather than policy solutions until the Isla Vista shooting. On the Monday after the Isla Vista shooting the *Los Angeles Times* ran an op-ed by Renée Binder, the incoming president for the American Psychiatric Association, expert on mental illness and violence, and California resident,. A key informant noted:

*That op-ed in the L.A. Times was so powerful. It was powerful because it was a complete lesson in media advocacy for me, where the theory of media advocacy is that media is not about information, it's about power, and, if you can control the narrative in the media, it doesn't really matter what the narrative says, you'll win the day. […] after that, the media narrative totally changed: “This is what we need to do. Why wouldn't we do this?” because people were like, “What are we going to do? Do we need better mental health laws? Do we need this? Do we need that?’”No, we need ERPO, and the L.A. Times laid that out there and everybody saw, and we spread it all over the place.*

Assembly Member Skinner proposed the GVRO bill on the first working day of the legislative session following the Isla Vista shooting, one day after the op-ed was published.

Key informants reported this policy window would not have yielded success if not for the preparation by advocates to draft the bill and soften up the proposal. According to one key informant:

*Well, the outreach really started well before the shooting on Memorial Day. When I teach this to my class, I say, “It appears that just a window opened and you just cobbled together.” It wasn't anything like that. We had a bill that wasn't done, but we were pretty confident that we had the right stakeholders, so really we were waiting on Assembly Member Skinner to introduce the bill. […] the bill was in good shape, so she could just drop it if she wanted to.*

One key informant estimated that, by 2014, lawmakers and firearm violence prevention advocates had spent about two years trying to raise awareness. While policy entrepreneurs had drafted an initial version of the GVRO bill prior to the Isla Vista shooting, they planned for a subsequent two-year effort to pass the law; the attack shifted the timeline to four months (including a one month Legislative recess that summer, during which no official legislative hearings could be held or bill amendments made). As one key informant stated “…the preparation that went into it was actually not quick, but our legislative strategy moved really fast.”

One strategy involved recruiting political entrepreneurs (i.e., legislators), including State Assembly Member Das Williams and State Senator Hannah Beth Jackson, representatives of Isla Vista. While the AB 1014 GVRO bill was initially a renewable energy program bill sponsored by Assembly Member Williams, Williams permitted Assembly Member Skinner to revise the bill in its entirety, allowing the GVRO legislation to work around deadlines and reducing the policy load faced by the legislature. A key informant reported, “It was very helpful to have the legislation drafted before it was needed. Having an effective multi-disciplinary team matters. Having buy-in from senior legislators who are thought leaders matters. To quote Rahm Emmanuel: ‘Never let a crisis go to waste.’”.

With the policy stream coupled with the problem stream in 2014, policy entrepreneurs shifted their attention to bargaining and decision-making in the politics stream.

### Politics stream

The broader political environment, including elections, public opinion, economics, and interest group lobbying, constitutes the politics stream. With respect to the GVRO bill, the politics stream largely involved negotiations and consensus building between advocates, legislators, and interest groups over the details of the proposal.

#### Bargaining

Because the bill was framed around dangerousness, including heightened anger, strain due to a significant loss, temporary despondence, and emotional trouble without a mental health diagnosis, policy entrepreneurs gained some support from mental health stakeholders. Nevertheless, the bill received pushback from the California Psychiatric Association. While a key informant who works as a psychiatrist initially supported the proposal to permit health care practitioners to petition for GVROs, discussions at a meeting at the capitol changed the informant’s mind:

*[…] there was somebody there who was like a malpractice attorney or, […] the attorney for the CMA [California Medical Association], who brought this perspective of “Once you have this tool available to you, you kind of have to use it, or you negligently didn't use it. And the way to get around that is to mandate it […].” And practitioners don’t want to be mandated to do things. Then are you just going to be filing one of these on everybody? It's going to be useless because it's so untailored. And so my mind really changed […].*

Once the legislature amended the bill to exclude therapists or licensed healthcare providers as eligible petitioners, the proposal gained support from the California Psychiatric Association. A key informant reported, “When you have the [California] Psychiatric Association and Disability Rights [California] supporting the bill, you know you’re in a good place,” noting that it was rare for the two organizations to support the same bill. Moreover, Disability Rights California’s support was particularly important as the support of the Pro Tempore of the Senate, who had close ties to the organization, was crucial to the success of the bill.

The potential for racial disparities in the implementation of GVROs represented another concern voiced by advocates, which informed the design of the policy. One key informant noted that GVROs were intentionally crafted to be non-criminalizing:

*Well, we also don't want to lock people up just because they're in crisis. We don't want to incarcerate or give law enforcement a tool that helps further incarcerate – particularly in communities of color where we see high disproportionate rates of incarceration. So, that is why the tool is not a – it's not creating new avenues for criminality. It's not a conviction.*

To address apprehensions that the tool may lead to incarceration, policy entrepreneurs designed the GVRO to be a civil process. Likewise, the bill includes family and household members as petitioners, due to concerns that communities distrustful of law enforcement may not otherwise use the policy:

*It is literally a civil process that is trying to deal specifically with the firearm and the individual at risk. And it's also why it doesn't solely rely in law enforcement. It's why family members are also included as the petitioners as well because communities that may have mistrust of law enforcement are more likely to engage with – or would be more likely to engage with the policy if they themselves could petition directly.*

Within the legislature, key informants identified due process and potential misuse as primary issues driving opposition to the bill. One advocate indicated that they modeled the ex parte order’s due process requirements after DVRO ex parte orders:

*There were questions about due process, but, once again, that is exactly why we modeled these after the domestic violence restraining order processes, and those are often [a] similar ex parte process. […] So, we feel – and continue to analyze and feel incredibly strongly – that these are constitutional, and they've been proven as such.*

Key informants identified the state Senate Public Safety hearing in June 2014 as a critical turning point. While advocates for the bill were uncertain of two committee members’ positions, and according to one key informant, “the Chair of the committee seemed disinclined to support the bill,” the testimony about the law’s potential utility by survivors, advocates, doctors, and law enforcement was “…so compelling that it passed out of committee. And it was a very emotional hearing.” This vote, and the years of efforts leading up to it, set the stage for the bill’s passage: “They flipped two votes and they were able to get that through and then, from then on, the bill had smooth sailing because of the effort around it and all the work that had been done to prepare for it.”

Several key informants cited the testimony by Ken James, Chief of the Emeryville Police Department. Per the Chief, the officers who contacted the shooter prior to the Isla Vista attack could not have legally conducted a search or recovered his firearms, even if they had knowledge of ownership. When asked why officers did not check the shooter’s firearm purchase history or search his apartment, the Chief stated that evidence of ownership does not permit law enforcement to “[…] look through someone’s personal possessions for a weapon simply because we know that weapon is there. […] there’s nothing in the laws, the statutes right now, that allows us to seize that weapon and to keep it from that individual’s hands.”

Key informants also identified Amanda Wilcox’s testimony as critical in persuading the Senate Public Safety Committee to support the GVRO. Wilcox lost her daughter to a mass shooting over a decade prior. Her testimony urged lawmakers to consider the temporary and reversible consequences of GVROs relative to those experienced by victims of firearm violence:

*And you know I usually try and wear my policy hat and be very reasonable. But I was really pushed over the edge by Richard Martinez, whose son was killed in Santa Barbara. And he said what about his son’s right to life. And I feel that about my daughter. What about her right? And I know we need to be careful about rights and mindful about due process but darn it we’re talking about life. And we can always give a firearm back. We cannot take a life back. I cannot get my daughter back.*

Subsequent to the Senate Public Safety Committee hearing, another key informant recounted concerns over misuse:

*There were obviously people who were really concerned about it being used to get revenge on other people. That was one of the main obstacles. They said, “Well, what if your neighbor and you are in a feud, and your neighbor just wants to take your guns away, and so he files a GVRO?” And it was really surprising to me how many people actually thought that this was going to be the thing that your nasty neighbor did to you, or your ex-wife […].*

To address concerns about misuse, advocates and lawmakers amended the bill to make it a misdemeanor crime to file a petition based on false information.

At this point, the politics stream was ripe; the majority party in both the State Senate and the Assembly moved to support a proposal on an issue the Democrats “owned”—firearm violence prevention [38]. After the Isla Vista shooting and the Senate Public Safety Committee hearing, values in the politics stream shifted such that imminent firearm violence risk was deemed a problem and the GVRO policy a solution.

#### Opposition

Law enforcement proved to be a persistent roadblock until just two months prior to the bill’s approval by the legislature; the policy idea had been tabled twice before, in 2003 and early 2014, due to law enforcement opposition. After a decade of resistance from law enforcement, Mendocino County Sheriff Tom Allman contacted Assembly Member Skinner and subsequently spoke in support of the bill at the California State Sheriff’s Association Meeting in July 2014. There, Sheriff Allman shared the story of his brother’s death by firearm suicide, which occurred after Allman tried unsuccessfully to obtain a psychiatric hold that would have prohibited possession of firearms. The Sheriff’s Association subsequently moved to support to the bill. One key informant said she “fell out of [her] chair” when she learned the Sheriff’s Association, typically opposed to firearm safety laws, made this change. She stated that “the broad support by organizations that often did not agree on policy was noteworthy and contributed to the passage of the bill. I often pointed out the ‘strange bedfellows’ when lobbying the bill.” This consensus-building constituted a major success in the decision window, further ripening the political stream.

The National Rifle Association (NRA) also posed opposition to the bill. Policy entrepreneurs strategically used the NRA’s own language to respond:

*The press release from the NRA came out maybe mid-summer, or maybe even August [2014], because the frame that we picked was, they’re always saying, “It's not the guns.” It's always, “Guns don't kill people. People kill people,” and we framed this as, “Okay. Who are the people? Let's just talk about that.”*

The diversity of stakeholders in the GVRO bill also enabled policy entrepreneurs to prepare a response to NRA opposition:

*I wanted to get everybody aligned so that if the NRA came in, they would be alone. I didn't want them picking off the consumer mental health group, or [the California] Psychiatric Association, or the ACLU [American Civil Liberties Union], […] we tried to get as many stakeholders as we could.*

#### Specialists

Early GVRO efforts in the policy and politics streams emphasized the importance of recruiting a range of advocates with specific roles, as policies that gain support from diverse specialists in policy networks increase the likelihood of adoption [37]. One key informant noted, “It was a well-orchestrated, unified front in support that anticipated what a lot of the barriers would be and were able to preempt them in some way.” The key informant went on to say:

*I think […] anticipating where the push-back is coming from and guiding the conversation or having your designated person for each concern or question. I knew it was my job to respond when somebody said, “Well, we have the §5150 for that.” I was like, “That's me!” […] But if somebody asked, “Well, why can't we prohibit misdemeanors?” that was [a colleague’s question], because he had done that research. So everyone knew what their voice was there for and what we were supposed to talk about.*

## Discussion

In this study, we applied Kingdon’s multiple streams framework to summarize the passage of California’s GVRO law, the first restraining-order-based ERPO law in the nation. Prior to this law, few mechanisms existed that permitted the temporary recovery of firearms from non-prohibited persons who made threats or behaved dangerously and had or could have access to a firearm.

A policy window opened in 2014 with the mass shooting in Isla Vista, and policy entrepreneurs quickly seized the opportunity. Owing to an iterative, years-long process involving numerous stakeholders, the GVRO bill was ready to be introduced to the state assembly immediately following the shooting. Policy entrepreneurs defended or modified the bill in the face of criticism from a variety of groups, and ultimately, through a combination of emotional anecdotes from survivors and scientific evidence, garnered enough support for the bill to be passed.

Consistent with other research [39], focusing events were integral to framing the problem and demonstrating the need for the GVRO law. Similar to the impact that the assassinations of Dr. Martin Luther King Jr. and Senator Robert Kennedy had on the Gun Control Act of 1968 [40], the 2014 mass shooting in Isla Vista, California was a focusing event in the problem stream and opened a policy window. Likewise, akin to the passage of legislation authorizing the Maryland State Superintendent of Police to create a list of handguns that could be legally sold in the state [41], academics and researchers strengthened the argument in favor of the GVRO bill by presenting evidence on the scope of firearm violence and the limitations of existing law.

Media coverage of mass shootings provided feedback to all streams throughout this process and proved to be both a complicating factor and a critical element to the policy’s success. Early on, reporting that framed mental illness as the primary cause of mass shootings contributed ambiguity to the problem’s framing. However, after the Isla Vista shooting, policy entrepreneurs capitalized on media as a platform to use emotional appeals and evidence to frame the GVRO law as the solution to the problem of imminent firearm violence by non-prohibited persons. Prior research has also found the media to play an important role in firearm violence legislation. One recent study examining media coverage of ERPOs in states that passed an ERPO law and those that considered, but did not pass, an ERPO law, found that news articles in passing states more frequently than in non-passing states used only official policy names for ERPOs (rather than referring to them as “red flag” laws), employed language about the prevention of access to firearms rather than the use of verbs such as “take away”, “seize”, and “remove”, mentioned gun violence prevention advocacy groups, cited research on ERPOs, and explicitly reported that a violent event was or could have been prevented by an ERPO [42]. Other work found that a single mass shooting is associated with a 15% increase in the number of firearm policies introduced in a state within a year of the shooting, and that the number of policies expands with increasing media coverage [43].

Policy entrepreneurs were persuaded by conversations with criminal justice reform experts to design the GVRO as a civil process to avoid creating new avenues of criminalization, which disproportionately affect persons of color [44–47]. Nevertheless, it is important to monitor for equity in implementation. An early-stage evaluation of the GVRO law revealed the racial/ethnic distribution of respondents to be largely reflective of the statewide firearm owning population [10]; however, Black respondents were the least likely to have legal representation in court for a long-term order after a hearing [11]. Likewise, while the policy was designed to broaden access by establishing family and household members as eligible petitioners in addition to law enforcement officers, 96.5% of GVRO petitioners between 2016 and 2018 were law enforcement officers [10]. No family or household members served as petitioners for Black or Latinx respondents [11]. People of color and members of other marginalized groups should be engaged in ERPO proposal development and enactment from the very beginning to help promote racial/ethnic equity in the design of the policy and its implementation.

This study is subject to limitations. We interviewed six key informants with direct involvement with the GVRO law’s creation, representing policy advocates and physician-researchers; findings may have differed with a larger sample size. Some strategies used by survivors of firearm violence who became policy advocates, such as lobbying or advocacy through the media, require social and financial capital not available to most of those who carry the disproportionate burden of firearm violence. While we believe we have identified a number of challenges and facilitators likely to generalize to similar laws and other states, we focused on a single case study in California, a state with a long history of regulating the possession and use of firearms, including policies passed in response to mass shootings. Policy entrepreneurs in other states may face different barriers and facilitators. However, since California passed its GVRO bill into law, 16 other states and the District of Columbia have enacted ERPO policies, indicating that California is not necessarily unique in its ability to pass ERPO legislation. Future research should conduct similar case studies in other states to determine differences and similarities.

## Conclusions

Kingdon describes policies as “an idea whose time has come” (p. 1) [18]. In the case of the GVRO law in California, a long history of relationship-building and softening up the proposal prepared policy entrepreneurs to capitalize on the policy window that opened after a focusing event in the problem stream—the Isla Vista, California mass shooting in 2014. As the recently enacted “Bipartisan Safer Communities Act” provides $750 million in incentives to states that implement crisis intervention services, including ERPO laws, results from this case study may inform other states seeking to implement such policies. Findings suggest that policymakers and advocates for firearm violence prevention should prepare extensively, develop bills early, cultivate and leverage relationships among policy entrepreneurs, engage diverse groups of stakeholders, recruit advocates affected by gun violence and those familiar with gaps in current law, anticipate long campaigns, and be prepared to take advantage of unexpected opportunities.

## Supplementary Information

**Additional file 1:** **Supplement Table 1.** Multiple Streams Concepts, Barriers, and Facilitators in the Passage of the GVRO law.

## Data Availability

The data generated and/or analysed during the current study are not publicly available due to privacy concerns, but de-identified data are available from the corresponding author on reasonable request.

## Abbreviations

ACLU: American Civil Liberties Union
DVRO: Domestic violence restraining order
ERPO: Extreme risk protection order
GVRO: Gun violence restraining order
NRA: National Rifle Association
