# Supplementary material for: The origins of California’s gun violence restraining order law: a case study using Kingdon’s multiple streams framework
Source: BMC Public Health. 2023 Jun 30;23:1275. doi: 10.1186/s12889-023-16043-6 (PMC10314549; doi:10.1186/s12889-023-16043-6)
Supplement: Supplementary file 1 — Additional file 1: Supplement Table 1. Multiple Streams Concepts, Barriers, and Facilitators in the Passage of the GVRO law. [file 12889_2023_16043_MOESM1_ESM.docx]

# Supplement

# Supplement Table 1. Multiple Streams Concepts, Barriers, and Facilitators in the Passage of the GVRO law

| **Multiple Streams Concept** | **Definition** | **Barrier** | **Facilitator** |
| --- | --- | --- | --- |
| Problem Stream | The social construction of the problem requiring political intervention | Problem frame ambiguity | Magnitude of the problem  Limitations of existing policies  Focusing events  Research |
| Policy Stream | Proposed ideas or policy solutions | Conveying the need for the GVRO law within the existing policy landscape | Long-term relationships among policy entrepreneurs  Forums engaging diverse stakeholders  GVROs as a targeted intervention that filled existing policy gaps  Logistic and economic feasibility |
| Policy Window | Moment in time where two streams merge, increasing the likelihood of adopting new policies | N/A | Focusing event  Media engagement by policy entrepreneurs  Survivor advocates  Previous outreach and bill drafting  Involvement of legislators |
| Politics Stream | The broader political environment, including elections, changes in public opinion, economics, and interest group lobbies | Concerns about unintended, harmful consequences  Resistance by the California Psychiatric Association to the eligibility of health care practitioners to serve as petitioners  Opposition to the bill by legislators over due process concerns  Opposition by law enforcement  Opposition by the NRA | GVRO criteria based on behavioral risk, not mental illness  Civil rather than criminal process  Misdemeanor crime to file libelous petition  Removal of health care practitioners from the list of eligible petitioners  Family members included as eligible petitioners to reduce reliance on law enforcement  Testimony and advocacy by persons affected by gun violence and experienced with the limitations of current law  Minimal opposition to law by firearm rights groups and use of the opposition’s language in framing of the bill |
